# Imitating Manual Curation of Text-Mined Facts in Biomedicine

**DOI:** 10.1371/journal.pcbi.0020118

**Published:** 2006-09-08

**Authors:** Raul Rodriguez-Esteban, Ivan Iossifov, Andrey Rzhetsky

**Affiliations:** 1 Department of Electrical Engineering, Columbia University, New York, New York, United States of America; 2 Center for Computational Biology and Bioinformatics, Joint Centers for Systems Biology, Columbia University, New York, New York, United States of America; 3 Department of Biomedical Informatics, Columbia University Medical Center, Columbia University, New York, New York, United States of America; 4 Judith P. Sulzberger, MD Columbia Genome Center, Columbia University Medical Center, Columbia University, New York, New York, United States of America; 5 Department of Biological Sciences, Columbia University, New York, New York, United States of America; Centro Nacional de Biotecnologia, Spain

## Abstract

Text-mining algorithms make mistakes in extracting facts from natural-language texts. In biomedical applications, which rely on use of text-mined data, it is critical to assess the quality (the probability that the message is correctly extracted) of individual facts—to resolve data conflicts and inconsistencies. Using a large set of almost 100,000 manually produced evaluations (most facts were independently reviewed more than once, producing independent evaluations), we implemented and tested a collection of algorithms that mimic human evaluation of facts provided by an automated information-extraction system. The performance of our best automated classifiers closely approached that of our human evaluators (ROC score close to 0.95). Our hypothesis is that, were we to use a larger number of human experts to evaluate any given sentence, we could implement an artificial-intelligence curator that would perform the classification job at least as accurately as an average individual human evaluator. We illustrated our analysis by visualizing the predicted accuracy of the text-mined relations involving the term *cocaine*.

## Synopsis

Current automated approaches for extracting biologically important facts from scientific articles are imperfect: while being capable of efficient, fast, and inexpensive analysis of enormous quantities of scientific prose, they make errors. To emulate the human experts evaluating the quality of the automatically extracted facts, we have developed an artificial intelligence program (“a robotic curator”) that closely approaches human experts in the quality of distinguishing the correctly extracted facts from the incorrectly extracted ones.

## Introduction

Information extraction uses computer-aided methods to recover and structure meaning that is locked in natural-language texts. The assertions uncovered in this way are amenable to computational processing that approximates human reasoning. In the special case of biomedical applications, the texts are represented by books and research articles, and the extracted meaning comprises diverse classes of facts, such as relations between molecules, cells, anatomical structures, and maladies.

**Figure 1:**
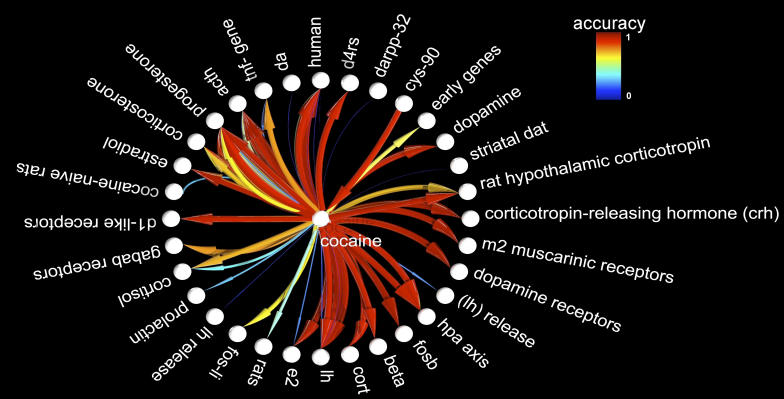
*Cocaine:* The Predicted Accuracy of Individual Text-Mined Facts Involving Semantic Relation *Stimulate* Each directed arc from an entity *A* to an entity *B* in this figure should be interpreted as a statement *“A stimulates B”,* where, for example, *A* is *cocaine* and *B* is *progesterone*. The predicted accuracy of individual statements is indicated both in color and in width of the corresponding arc. Note that, for example, the relation between *cocaine* and *progesterone* was derived from multiple sentences, and different instances of extraction output had markedly different accuracy. Altogether we collected 3,910 individual facts involving *cocaine*. Because the same fact can be repeated in different sentences, only 1,820 facts out of 3,910 were unique. The facts cover 80 distinct semantic relations, out of which *stimulate* is just one example.

Unfortunately, the current tools of information extraction produce imperfect, noisy results. Although even imperfect results are useful, it is highly desirable for most applications to have the ability to rank the text-derived facts by the confidence in the quality of their extraction (as we did for relations involving *cocaine*, see Figure 1). We focus on automatically extracted statements about molecular interactions, such as *small molecule A binds protein B*, *protein B activates gene C*, or *protein D phosphorylates small molecule E*. (In the following description we refer to phrases that represent biological entities—such as *small molecule A*, *protein B*, and *gene C—*as *terms*, and to biological relations between these entities—such as *activate* or *phosphorylate—*as *relations* or *verbs*.)

Several earlier studies have examined aspects of evaluating the quality of text-mined facts. For example, Sekimizu et al. and Ono et al. attempted to attribute different confidence values to different verbs that are associated with extracted relations, such as *activate, regulate,* and *inhibit* [2,3]. Thomas et al. proposed to attach a quality value to each extracted statement about molecular interactions [4], although the researchers did not implement the suggested scoring system in practice. In an independent study [5], Blaschke and Valencia used word-distances between biological terms in a given sentence as an indicator of the precision of extracted facts. In our present analysis we applied several machine-learning techniques to a large training set of 98,679 manually evaluated examples (pairs of extracted facts and corresponding sentences) to design a tool that mimics the work of a human curator who manually cleans the output of an information-extraction program.

### Approach

Our goal is to design a tool that can be used with any information-extraction system developed for molecular biology. In this study, our training data came from the GeneWays project (specifically, GeneWays 6.0 database [6,7]), and thus our approach is biased toward relationships that are captured by that specific system (Text S1 Note 1). We believe that the spectrum of relationships represented in the GeneWays ontology is sufficiently broad that our results will prove useful for other information-extraction projects.

Our approach followed the path of supervised machine-learning. First, we generated a large training set of facts that were originally gathered by our information-extraction system, and then manually labeled as “correct” or “incorrect” by a team of human curators. Second, we used a battery of machine-learning tools to imitate computationally the work of the human evaluators. Third, we split the training set into ten parts, so that we could evaluate the significance of performance differences among the several competing machine-learning approaches.

## Methods

### Training data

With the help of a text-annotation company, ForScience, we generated a training set of approximately 45,000 multiply annotated unique facts, or almost 100,000 independent evaluations. These facts were originally extracted by the GeneWays pipeline, then were annotated by biology-savvy doctoral-level curators as “correct” or “incorrect,” referring to quality of information extraction. Examples of automatically extracted relations, sentences corresponding to each relation, and the labels provided by three evaluators are shown in Table 1.

**Table 1:**
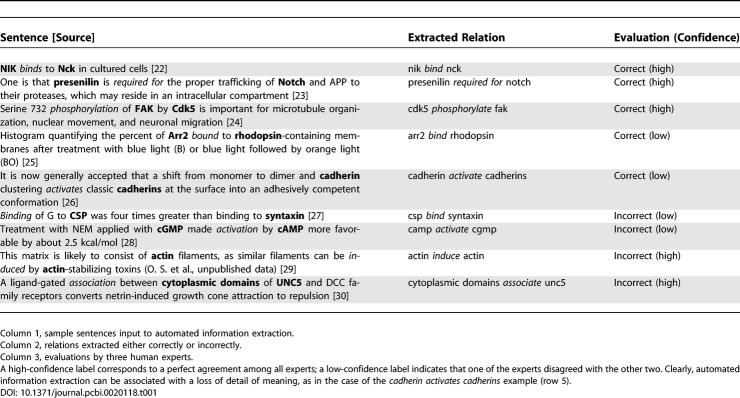
A Sample of Sentences That Were Used as an Input to Automated Information Extraction, Biological Relations Extracted from These Sentences, and the Corresponding Evaluations

Each extracted fact was evaluated by one, two, or three different curators. The complete evaluation set comprised 98,679 individual evaluations performed by four different people, so most of the statement–sentence pairs were evaluated multiple times, with each person evaluating a given pair at most once. In total, 13,502 statement–sentence pairs were evaluated by just one person, 10,457 by two people, 21,421 by three people, and 57 by all four people. Examples of both high inter-annotator agreement and low-agreement sentences are shown in Table 1.

The statements in the training dataset were grouped into chunks; each chunk was associated with a specific biological project, such as analysis of interactions in Drosophila melanogaster. Pairwise agreement between evaluators was high (92%) in most chunks (Text S1 Note 2), with the exception of a chunk of 5,271 relations where agreement was only 74%. These relatively low-agreement evaluations were not included in the training data for our analysis (Text S1 Note 3).

To facilitate evaluation, we developed a Sentence Evaluation Tool implemented in Java programming language by Mitzi Morris and Ivan Iossifov. This tool presented to an evaluator a set of annotation choices regarding each extracted fact; the choices are listed in Table 2. The tool also presented in a single window the fact itself and the sentence it was derived from. In the case that a broader context was required for the judgement, the evaluator had a choice to retrieve the complete journal article containing this sentence by clicking a single button on the program interface.

**Table 2:**
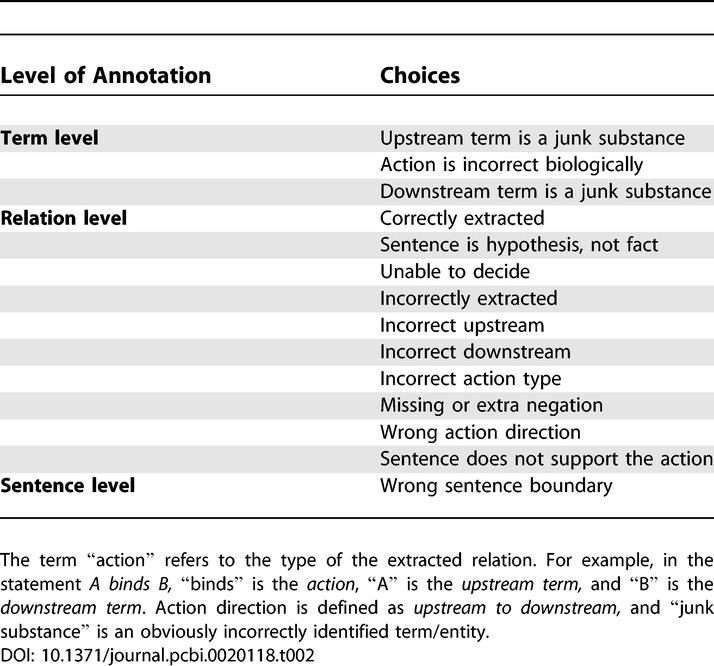
List of Annotation Choices Available to the Evaluators

For the convenience of representing the results of manual evaluation, we computed an evaluation score for each statement as follows. Each sentence–statement score was computed as a sum of the scores assigned by individual evaluators; for each evaluator, −1 was added if the expert believed that the presented information was extracted incorrectly, and +1 was added if he or she believed that the extraction was correct. For a set of three experts, this method permitted four possible scores: 3(1,1,1), 1(1,1,−1), −1(1,−1, −1), and −3. Similarly, for just two experts, the possible scores are 2(1,1), 0(1,−1), and −2(−1,−1) (Text S1 Note 4).

### Computational Methods

#### Machine-learning algorithms: General framework.

The objects that we want to classify, the fact–sentence pairs, have complex properties. We want to place each of the objects into one of two classes, *correct* or *incorrect*. In the training data, each extracted fact is matched to a unique sentence from which it was extracted, even though multiple sentences can express the same fact and a single sentence can contain multiple facts. The *i* ^th^ object (the *i* ^th^ fact–sentence pair) comes with a set of known features or properties that we encode into a feature vector, **F** *_i_*:

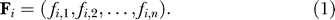



In the following description we use *C* to indicate the random variable that represents class (with possible values *c_correct_* and *c_incorrect_*), and *F* to represent a 1 × *n* random vector of feature values (also often called *attributes*), such that *F_j_* is the *j* ^th^ element of *F*. For example, for fact *p53 activates JAK,* feature *F*_1_ would have value 1 because the upstream term *p53* is found in a dictionary derived from the GenBank database [8]; otherwise, it would have value 0.

#### Full Bayesian inference.

The full Bayesian classifier assigns the *i* ^th^ object to the *k* ^th^ class if the posterior probability *P*(*C* = *c_k_* | *F* = **F** *_i_*) is greater for the *k* ^th^ class than for any alternative class. This posterior probability is computed in the following way (a restated version of Bayes' theorem).

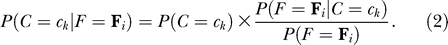



In the real-life applications, we estimate probability *P*(*F* = **F** *_i_* | *C* = *c_k_*) from the training data as a ratio of the number of objects that belong to the class *c_k_ and* have the same set of feature values as specified by the vector **F** *_i_* to the total number of objects in class *c_k_* in the training data.

In other words, we estimate the conditional probability for every possible value of the feature vector *F* for every value of class *C*. Assuming that all features can be discretized, we have to estimate

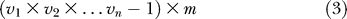
parameters, where *v_i_* is the number of discrete values observed for the *i* ^th^ feature and *m* is the number of classes.

Clearly, even for a space of only 20 binary features (Text S1 Note 5), the number of parameters that we would need to estimate is (2^20^ −1) × 2 = 2,097,150, which exceeds several times the number of datapoints in our training set.

#### Naïve Bayes classifier.

The most affordable approximation to the full Bayesian analysis is the Naïve Bayes classifier. It is based on the assumption of conditional independence of features:

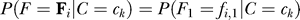


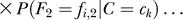


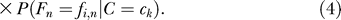



Obviously, we can estimate *P*(*F_j_* = *f_i,j_* | *C* = *c_k_*)s reasonably well with a relatively small set of training data, but the assumption of conditional independence (Equation 4) comes at a price: the Naïve Bayes classifier is usually markedly less successful in its job than are its more sophisticated relatives (Text S1 Note 6).

In an application with *m* classes and *n* features (given that the *i* ^th^ feature has *v_i_* admissible discrete values), a Naïve Bayes algorithm requires estimation of *m* × Σ*_i=1_*_,*n*_ (*v_i_* − 1) parameters (which value, in our case, is equal to 4,208).

### Middle Ground between the Full and Naïve Bayes: Clustered Bayes

We can find an intermediate ground between the full and Naïve Bayes classifiers by assuming that features in the random vector *F* are arranged into groups or clusters, such that all features within the same cluster are dependent on one another (conditionally on the class), and all features from different classes are conditionally independent. That is, we can assume that the feature random vector (*F*) and the observed feature vector for the *i* ^th^ object (**F** *_i_*) can be partitioned into subvectors:

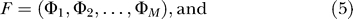


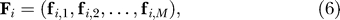
respectively, where Φ*_j_* is the *j* ^th^ cluster of features; **f** *_i,j_* is the set of values for this cluster with respect to the *i* ^th^ object, and *M* is the total number of clusters of features.

The Clustered Bayes classifier is based on the following assumption about conditional independence of *clusters* of features:

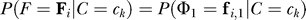


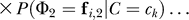


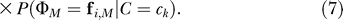



**Figure 2:**
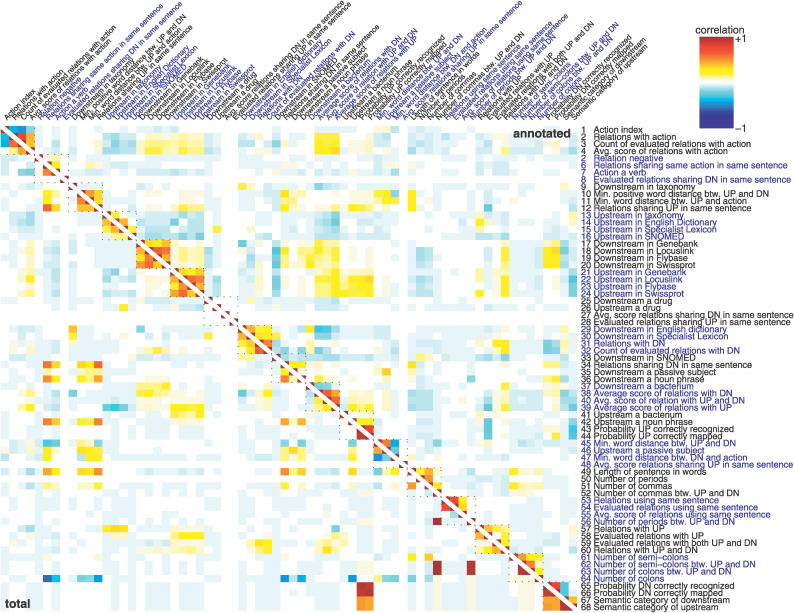
The Correlation Matrix for the Features Used by the Classification Algorithms The half-matrix below the diagonal was derived from analysis of the whole GeneWays 6.0 database; the half-matrix above the diagonal represents a correlation matrix estimated from only the manually annotated dataset. The white dotted lines outline clusters of features, suggested by analysis of the annotated dataset; we used these clusters in implementation of the Clustered Bayes classifier. We used two versions of the Clustered Bayes classifier: with all 68 features (Clustered Bayes 68), and with a subset of only 44 features but a higher number of discrete values allowed for nonbinary features (Clustered Bayes 44). The Clustered Bayes 44 classifier did not use features 1, 6, 7, 8, 9, 12, 27, 28, 31, 34, 37, 40, 42, 47, 48, 49, 52, 54, 55, 60, 62, 63, and 65.

We tested two versions of the Clustered Bayes classifier: one version used all 68 features (Clustered Bayes 68) with a coarser discretization of feature values; another version used a subset of 44 features (Clustered Bayes 44) but allowed for more discrete values for each continuous-valued feature, see legend to Figure 2.

### Linear and Quadratic Discriminants

Another method that can be viewed as an approximation to full Bayesian analysis is Discriminant Analysis invented by Sir Ronald A. Fisher [9]. This method requires no assumption about conditional independence of features; instead, it assumes that the conditional probability *P* (*F* = **F** *_i_* | *C* = *c_k_*) is a multivariate normal distribution.

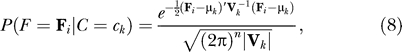
where *n* is the total number of features/variables in the class-specific multivariate distributions. The method has two variations. The first, *Linear Discriminant Analysis,* assumes that different classes have different mean values for features (vectors *μ_k_*), but the same variance–covariance matrix, **V** = **V** *_k_* for all *k* (Text S1 Note 7). In the second variation, *Quadratic Discriminant Analysis* (QDA), the assumption of the common variance–covariance matrix for all classes, is relaxed, such that every class is assumed to have a distinct variance–covariance matrix, **V** *_k_* (Text S1 Note 8).

In this study we present results for QDA; the difference from the linear discriminant analysis was insignificant for our data (unpublished data). In terms of the number of parameters to estimate, QDA uses only two symmetrical class-specific covariance matrices and the two class-specific mean vectors. For 68 features the method requires estimation of 2 × (68 × 69)/2 + 2 × 68 = 4,828 parameters.

#### Maximum-entropy method.

The current version of the maximum-entropy method was formulated by E. T. Jaynes [10,11]; the method can be traced to earlier work by J. Willard Gibbs. The idea behind the approach is as follows. Imagine that we need to estimate a probability distribution from an incomplete or small dataset—this problem is the same as that of estimating the probability of the class given the feature vector *P* (*C* = *c_k_* | *F* = **F** *_i_*), from a relatively small training set. Although we have no hope of estimating the distribution completely, we can estimate with sufficient reliability the first (and, potentially, the second) moments of the distribution. Then, we can try to find a probability distribution that has the same moments as our unknown distribution and the highest possible Shannon's entropy—the intuition behind this approach being that the maximum-entropy distribution will minimize unnecessary assumptions about the unknown distribution. The maximum-entropy distribution with constraints imposed by the first-order feature moments alone (the mean values of features) is known to have the form of an exponential distribution [12]:

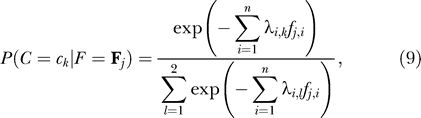
and the maximum-entropy distribution for the case when both the first-order and the second-order moments of the unknown distribution are fixed has the form of a multidimensional normal distribution [12]. The conditional distribution that we are trying to estimate can be written in the following exponential form:

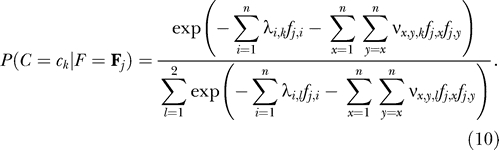



Parameters *λ_i,k_* and *v_x,y,k_* are *k*-class–specific weights of individual features and feature pairs, respectively, and in principle can be expressed in terms of the first and second moments of the distributions. The values of parameters in Equations 9and 10 are estimated by maximizing the product of probabilities for the individual training examples.

We tested two versions of the maximum-entropy classifier. MaxEnt 1 uses only information about the first moments of features in the training data (Equation 9); MaxEnt 2 uses the set of all individual features and the products of feature pairs (Equation 10). To select the most informative pairs of features, we used a mutual information approach, as described in the subsection dealing with classification features.

For two classes *(correct* and *incorrect)* and 68 features, MaxEnt 1 requires estimation of 136 parameters. In contrast, MaxEnt 2 requires estimation of 4,828 parameters: weight parameters for all first moments for two classes, plus weights for the second moments for two classes. MaxEnt 2-v is a version of MaxEnt 2 classifier where the squared values of features are not used, so that the classifier requires estimation of only 4,692 weight parameters.

### Feed-Forward Neural Network

**Figure 3:**
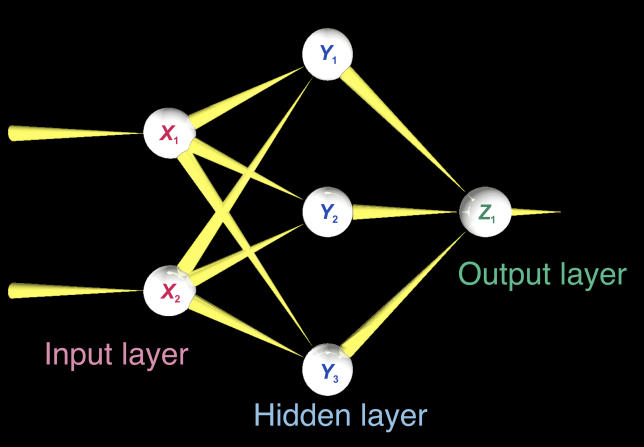
A Hypothetical Three-Layered Feed-Forward Neural Network We used a similar network with 68 input units (one unit per classification feature) and ten hidden-layer units.

A typical feed-forward artificial neural network is a directed acyclic graph organized into three (or more) layers. In our case, we chose a three-layered network, with a set of nodes of the *input layer*, {*x_i_*}*_i=_*_1,…,*N_x_*_, nodes of the *hidden layer*, {*y_j_*}*_j=_*_1,…,*N_y_*_, and a single node representing the *output layer*, *z*_1_, see Figure 3. The number of input nodes, *N_x,_* is determined by the number of features used in the analysis (68 in our case). The number of hidden nodes, *N_y,_* determines both the network's expressive power and its ability to generalize. Too small a number of hidden nodes makes a simplistic network that cannot learn from complex data. Too large a number makes a network that tends to overtrain—that works perfectly on the training data, but poorly on new data. We experimented with different values of *N_y_* and settled on *N_y_* = 10.

The values of the input nodes, {*x_i_*}*_i=_*_1,…,N_*x*_,_ are feature values of the object that we need to classify. The value of each node, *y_j_,* in the hidden layer is determined in the following way:

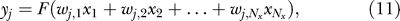
where *F*(*x*) is a hyperbolic tangent function that creates an S-shaped curve:

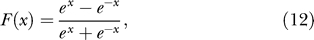
and {*w_j,k_*} are weight parameters. Finally, the value of the output node *z*_1_ is determined as a linear combination of the values of all hidden nodes:

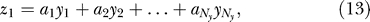
where {*a_k_*} are additional weight parameters. We trained our network, using a back-propagation algorithm [13], to distinguish two classes, *correct* and *incorrect,* where positive values of *z*_1_ corresponded to the class *correct*. The feed-forward neural network that we used in our analysis can be thought of as a model with *N_x_* × *N_y_* + *N_y_* parameters (690 in our case).

### Support Vector Machines

The Support Vector Machines (SVM, [14,15]) algorithm solves a binary classification problem by dividing two sets of data geometrically, by finding a hyperplane that separates the two classes of objects in the training data in an optimum way (maximizing the margin between the two classes).

The SVM is a *kernel*-based algorithm, where the kernel is an inner product of two feature vectors (function/transformation of the original data). In this study, we used three of the most popular kernels: the linear, polynomial, and Rbf (radial basis function) kernels. The linear kernel *K* ^L^ (*x*_1_,*x*_2_) = 〈*x*_1_,*x*_2_〉 is simply the inner product of the two input feature vectors; an SVM with the linear kernel searches for a class-separating hyperplane in the original space of the data. Using a polynomial kernel, 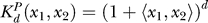 , is equivalent to transforming the data into a higher-dimensional space and searching for a separating plane there (Text S1 Note 9). Finally, using an Rbf kernel, 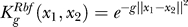 , corresponds to finding a separating hyperplane in an infinite-dimensional space.

In the most real-world cases the two classes cannot be separated perfectly by a hyperplane, and some classification errors are unavoidable. SVM algorithms use the *C*-parameter to control the error rate during the training phase (if the error is not constrained, the margin of every hyperplane can be extended infinitely). In this study, we used the default values for the *C*-parameter suggested by the SVM Light tool. Table 3 lists the SVM models and *C*-parameter values that we used in this study.

**Table 3:**
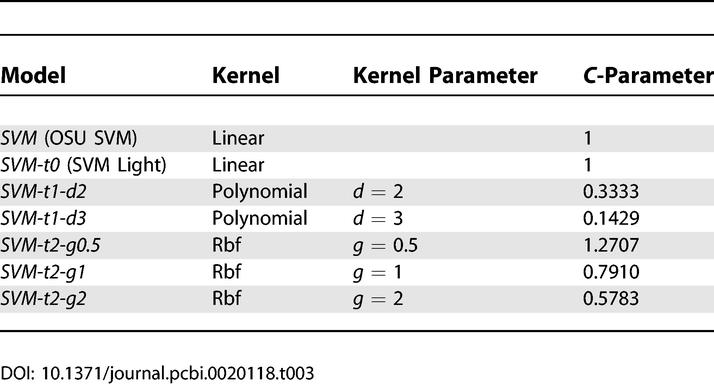
Parameter Values Used for Various SVM Classifiers in This Study

The output of an SVM analysis is not probabilistic, but there are tools to convert an SVM classification output into “posterior probabilities” (see chapter by J. Platt in [16]). (A similar comment is applicable to the artificial neural network.)

The number of support vectors used by the SVM classifier depends on the size and properties of the training dataset. The average number of (1 × 68-dimensional) support vectors used in ten cross-validation experiments was 12,757.5, 11,994.4, 12,092, 12,289.9, 12,679.7, and 14,163.8, for SVM, SVM-t1-d2, SVM-t1-d3, SVM-t2-g0.5, SVM-t2-g1, and SVM-t2-g2 classifiers, respectively. The total number of data-derived values (which we loosely call “parameters”) used by the SVM in our cross-validation experiments was therefore, on average, between 827,614 and 880,270 for various SVM versions.

#### Meta-method.

We implemented the meta-classifier on the basis of the SVM algorithm (linear kernel with *C =* 1) applied to predictions (converted into probabilities that the object belongs to the class *correct*) provided by the individual “simple” classifiers. The meta-method used 1,445 support vectors (1 × 7-dimensional), in addition to combined parameters of the seven individual classifiers used as input to the meta-classifier.

#### Implementation.

A summary of the sources of software used in our study is shown in Table 4.

**Table 4:**
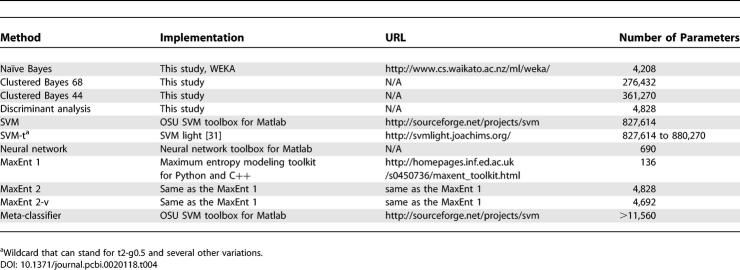
Machine Learning Methods Used in This Study and Their Implementations

### Features Used in Our Analysis

We selected 68 individual features covering a range of characteristics that could help in the classification (see Table 5). To capture the flow of information in a molecular interaction graph (the edge direction) in each extracted relation we identified an “upstream term” (corresponding to the graph node with the outgoing directed edge) and a “downstream term” (the node with the incoming directed edge): for example, in the phrase *“JAK* phosphorylates *p53*,” *JAK* is the upstream term, and *p53* is the downstream term. Features in the group *keywords* represent a list of tokens that may signal that the sentence is hypothetical, interrogative, negative, or that there is a confusion in the relation extraction (e.g., the particle “by” in passive-voice sentences). We eventually abandoned *keywords* as we found them to be uninformative features, but they are still listed for the sake of completeness.

**Table 5:**
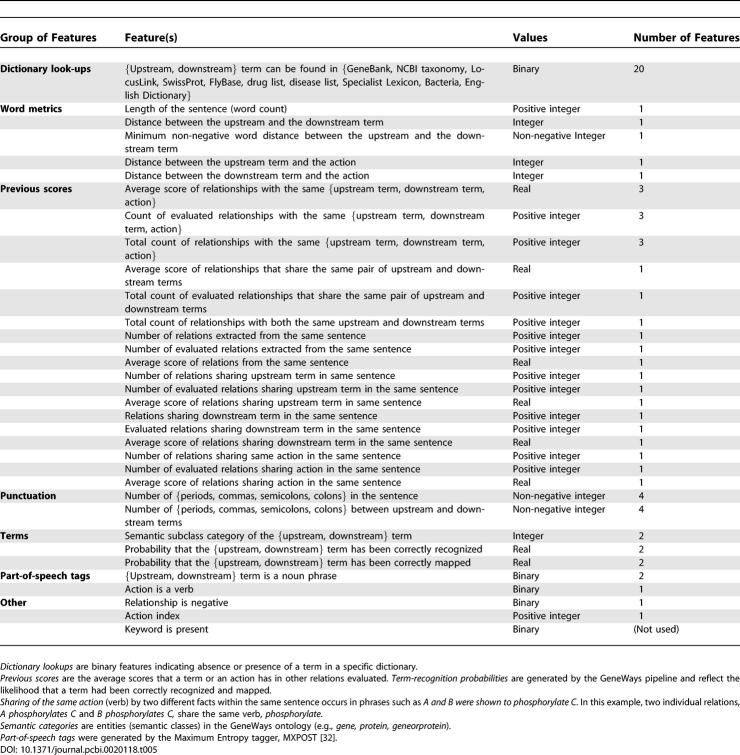
List of the Features That We Used in the Present Study

To represent the second-order features (pairs of features), we defined a new feature as a product of the normalized values of two features. We obtained the normalized values of features by subtracting the mean value from each feature value, then dividing the result by the standard deviation for this feature.

After a number of feature-selection experiments for the MaxEnt 2 method, we settled on using *all* second-order features.

### Separating Data into Training and Testing: Cross-Validation

To evaluate the success of our classifiers we used a 10-fold cross-validation approach, where we used 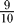 of data for training and 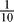 for testing. More precisely, given a partition of the manually evaluated data into ten equal portions, we created ten different pairs of training–test subsets, so that ten distinct testing sets put together covered the whole collection of the manually evaluated sentences. We then used ten training–test-set pairs to compare all algorithms.

### Comparison of Methods: ROC Scores

To quantify and compare success of the various classification methods, we used receiver operating characteristic (ROC) scores, also called *areas under ROC curve* [17].

**Figure 4:**
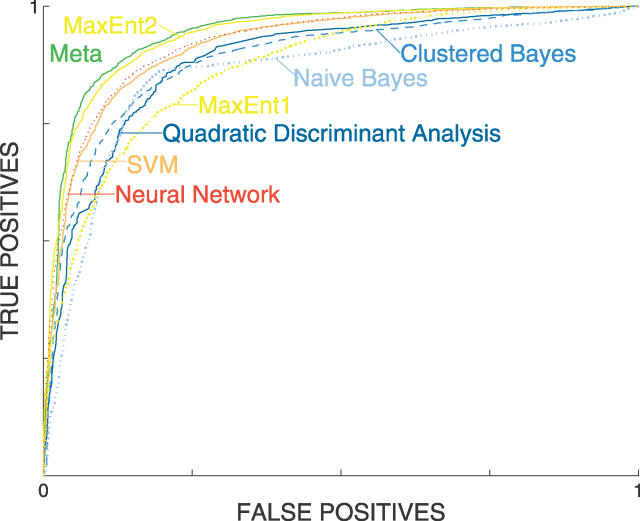
ROC Curves for the Classification Methods Used in the Present Study We show only the linear-kernel SVM and the Clustered Bayes 44 ROC curves to avoid excessive data clutter.

An ROC score is computed in the following way. All test-set predictions of a particular classification method are ordered by the decreasing quality score provided by this method; for example, in the case of the Clustered Bayes algorithm, the quality score is the posterior probability that the test object belongs to the class *correct*. The ranked list is then converted into binary predictions by applying a decision threshold, *T:* all test objects with a quality score above *T* are classified as *correct,* and all test objects with lower-than-threshold scores are classified as *incorrect*. The ROC score is then computed by plotting the proportion of true-positive predictions (in the test set we know both the correct label and the quality score of each object) against false-positive predictions for the whole spectrum of possible values of *T,* then integrating the area under the curve obtained in this way, see Figure 4.

The ROC score is an estimate of the probability that the classifier under scrutiny will label correctly a pair of statements, one of which is from the class *correct* and one from the class *incorrect* [17]. A completely random classifier therefore would have an ROC score of 0.5, whereas a hypothetical perfect classifier would have an ROC score of 1. It is also possible to design a classifier that performs less accurately than would one that is completely random; in this case the ROC score is less than 0.5, which indicates that we can improve the accuracy of the classifier by simply reversing all predictions.

## Results

**Figure 5:**
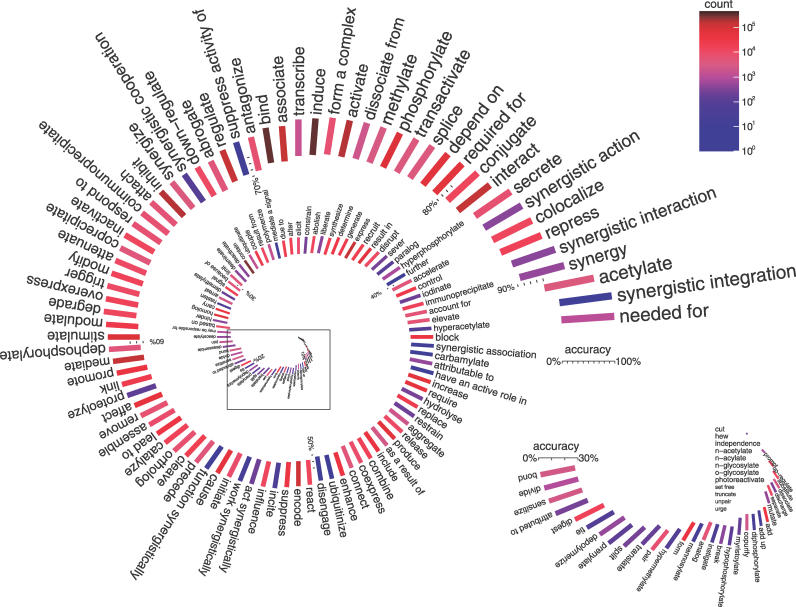
Accuracy of the Raw (Noncurated) Extracted Relations in the GeneWays 6.0 Database The accuracy was computed by averaging over all individual specific information extraction examples manually evaluated by the human curators. The plot compactly represents both the per-relation accuracy of the extraction process (indicated with the length of the corresponding bar) and the abundance of the corresponding relations in the database (represented by the bar color). There are relations extracted with a high precision; there are also many noisy relationships. The database accuracy was markedly increased by the automated curation outlined in this study (see Figure 9).

The raw extracted facts produced by our system are noisy. Although many relation types are extracted with accuracy above 80%, and even above 90% (see Figure 5), there are particularly noisy verbs/relations that bring the average accuracy of the “raw” data to about 65% (Text S1 Note 10). Therefore, additional purification of text-mining output, either computational or manual, is indeed important.

The classification problem of separating correctly and incorrectly extracted facts appears to belong to a class of easier problems. Even the simplest Naïve Bayes method had an average ROC score of 0.84, which more sophisticated approaches surpassed to reach almost 0.95. Judging by the average ROC score, the quality of prediction increased in the following order of methods: Clustered Bayes 68, Naïve Bayes, MaxEnt 1, Clustered Bayes 44, QDA, artificial neural network, SVMs, and MaxEnt 2/MaxEnt 2-v (see Table 6). The Meta-method was always slightly more accurate than MaxEnt 2, as explained in the legend to Table 6 and as shown in Figure 4.

**Table 6:**
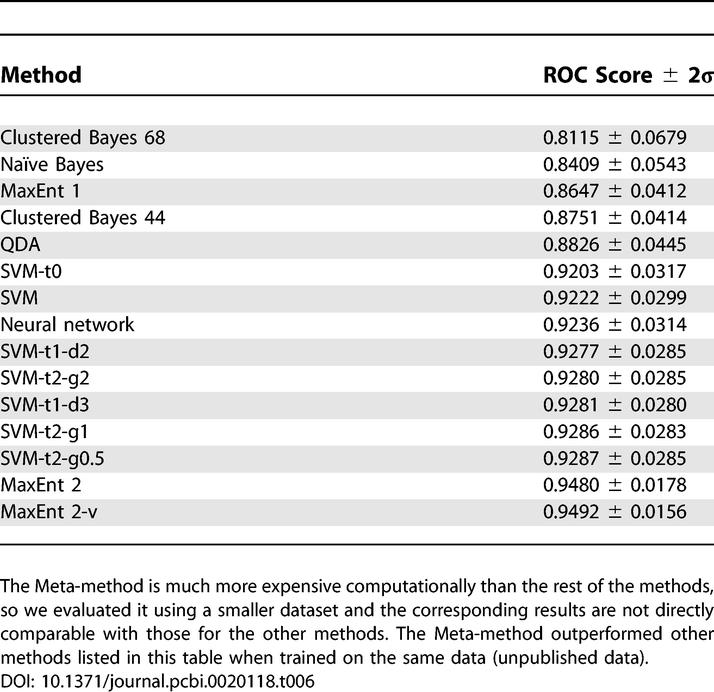
ROC Scores for Methods Used in This Study, with Error Bars Calculated in 10-Fold Cross-Validation

**Figure 6:**
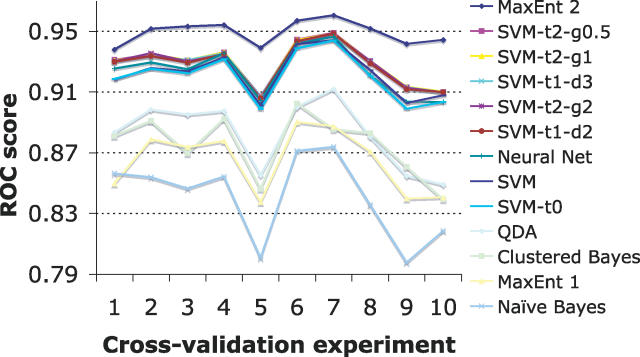
Ranks of All Classification Methods Used in This Study in Ten Cross-Validation Experiments

Table 6 provides a somewhat misleading impression that MaxEnt 2 and MaxEnt 2-v are *not* significantly more accurate than their closest competitors (the SVM family), because of the overlapping confidence intervals. However, when we trace the performance of all classifiers in individual cross-validation experiments (see Figure 6) it becomes clear that MaxEnt 2 and MaxEnt 2-v outperformed their rivals in every cross-validation experiment. The SVM and artificial neural network methods performed essentially identically, and were always more accurate than three other methods: QDA, Clustered Bayes 44, and MaxEnt 1. Finally, the performance of the Clustered Bayes 68 and the Naïve Bayes methods was reliably the least accurate of all methods studied.

It is a matter of both academic curiosity and of practical importance to know how the performance of our artificial intelligence curator compares with that of humans. If we define the *correct* answer as a majority-vote of the three human evaluators (see Table 6), the average accuracy of MaxEnt 2 is slightly lower than, but statistically indistinguishable from, humans (at the 99% level of significance, see Table 6; capital letters “A,” “L,” “S,” and “M” hide the real names of the human evaluators). If, however, in the spirit of Turing's test of machine intelligence [18], we treat the MaxEnt 2 algorithm on an equal footing with the human evaluators, compute the average over predictions of all four anonymous evaluators, and compare the quality of the performance of each evaluator with regard to the average, MaxEnt 2 always performs slightly more accurately (Text S1 Note 11) than one of the human evaluators (Text S1 Note 12). (In all cases we compared performance of the algorithm on data that was not used for its training; see Tables 6 and 7.)

**Table 7:**
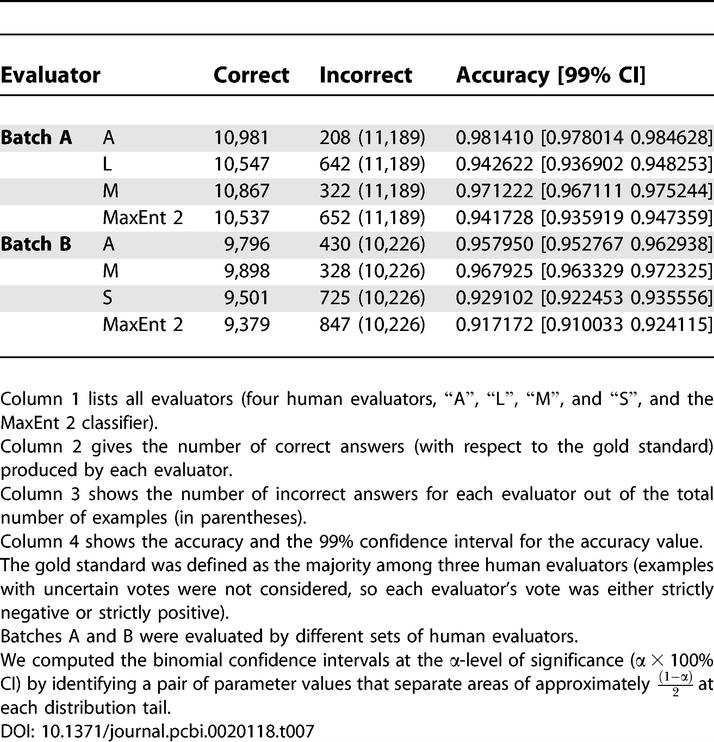
Comparison of the Performance of Human Evaluators and of the MaxEnt 2 Algorithm

**Figure 7:**
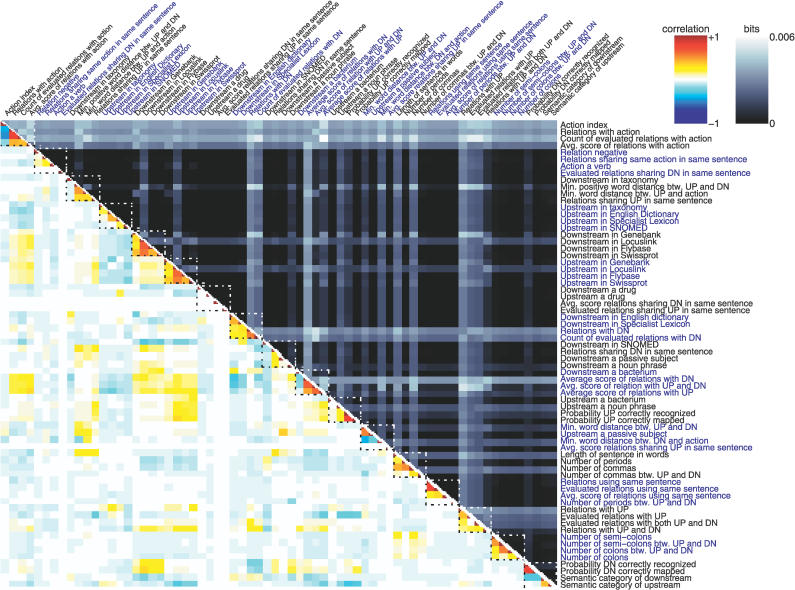
Comparison of a Correlation Matrix for the Features (Colored Half of the Matrix) Computed Using Only the Annotated Set of Data and a Matrix of Mutual Information between All Feature Pairs and the Statement Class (Correct or Incorrect) The plot indicates that a significant amount of information critical for classification is encoded in pairs of weakly correlated features. The white dotted lines outline clusters of features, suggested by analysis of the annotated dataset; we used these clusters in implementation of the Clustered Bayes classifier.

The features that we used in our analysis are obviously not all equally important. To elucidate the relative importance of the individual features and of feature pairs, we computed the mutual information between all pairs of features and the class variable (see Figure 7). The mutual information of class variable *C,* and a pair of feature variables (*F_i_,F_j_*), is defined in the following way (e.g., see [19]).

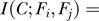


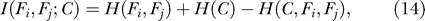
where function *H*(*P*[*x*]) is Claude E. Shannon's entropy of distribution *P*(*x*) (see p. 14 of [20]), defined in the following way:

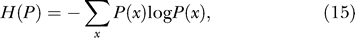
where summation is done over all admissible values of *x*. Figure 7 shows that the most informative standalone features, as expected, are those that are derived from the human evaluations of the quality of extraction of individual relations and terms (such as the average quality scores), and features reflecting properties of the sentence that was used to extract the corresponding fact. In addition, some dictionary-related features, such as finding a term in LocusLink, are fairly informative. Some features, however, become informative only in combination with other features. For example, the minimum positive distance between two terms in a sentence is not very informative by itself, but becomes fairly useful in combination with other features, such as the number of commas in the sentence or the length of the sentence (see Figure 7). Similarly, while finding a term in GenBank does not help the classifier by itself, the feature becomes informative in combination with syntactic properties of the sentence and statistics about the manually evaluated data.

**Figure 8:**
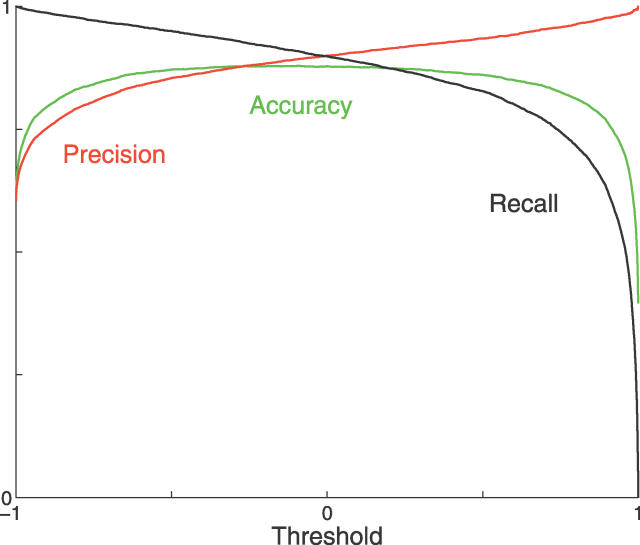
Values of Precision, Recall, and Accuracy of the MaxEnt 2 Classifier Plotted against the Corresponding Log-Scores Provided by the Classifier Precision is defined as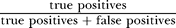 , recall is defined as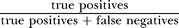 , and accuracy is defined as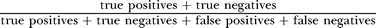 . The optimum accuracy was close to 88%, and attained a score threshold slightly above 0. We can improve precision at the expense of accuracy. For example, by setting the threshold score to 0.6702, we can bring the overall database precision to 95%, which would correspond to a recall of 77.91% and to an overall accuracy of 84.18%.

Assignment of facts to classes *correct* and *incorrect* by evaluators is subject to random errors: facts that were seen by many evaluators would be assigned to the appropriate class with higher probability than facts that were seen by only one evaluator. This introduction of noise directly affects the estimate of the accuracy of an artificial intelligence curator: if the gold standard is noisy, the apparent accuracy of the algorithm compared with the gold standard is lower than the real accuracy. Indeed, the three-evaluator gold standard (see Table 8) indicated that the actual optimum accuracy of the MaxEnt 2 classifier is higher than 88%. (The 88% accuracy estimate came from comparison of MaxEnt 2 predictions with the whole set of annotated facts, half of which were seen by only one or two evaluators; see Figure 8.) When MaxEnt 2 was compared with the three-human gold standard, the estimated accuracy was about 91% (see Table 8).

**Table 8:**
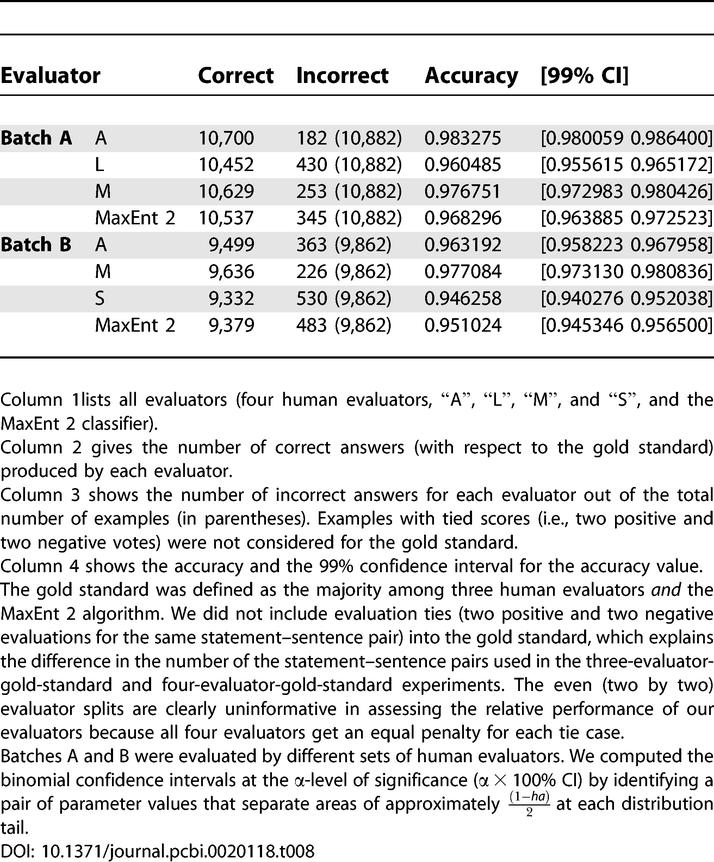
Comparison of Human Evaluators and a Program That Mimicked Their Work

## Discussion

**Figure 9:**
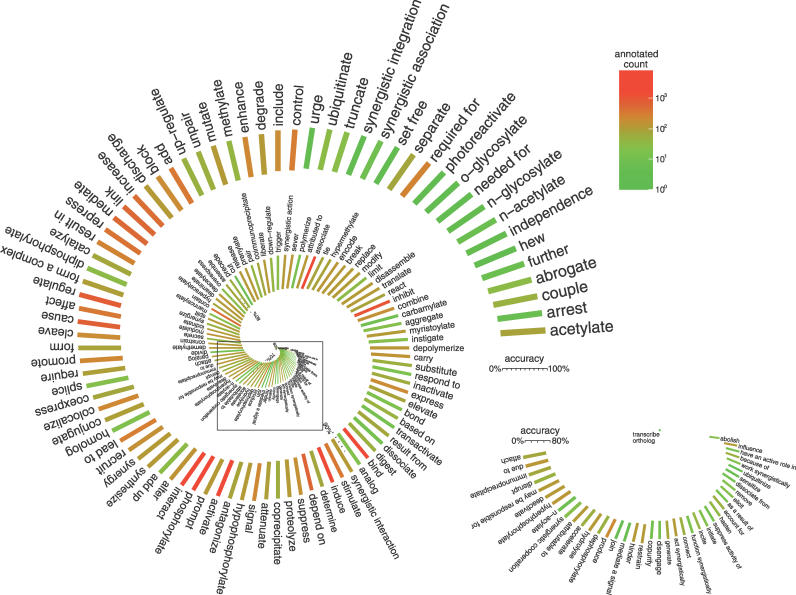
Accuracy and Abundance of the Extracted and Automatically Curated Relations This plot represents both the per-relation accuracy after both information extraction and automated curation were done. Accuracy is indicated with the length of the relation-specific bars, while the abundance of the corresponding relations in the manually curated dataset is represented by color. Here, the MaxEnt 2 method was used for the automated curation. The results shown correspond to a score-based decision threshold set to zero; that is, all negative-score predictions were treated as “incorrect.” An increase in the score-based decision boundary can raise the precision of the output at the expense of a decrease in the recall (see Figure 8).

As evidenced by Figures 5 and 9, the results of our study are directly applicable to analysis of large text-mined databases of molecular interactions: we can identify sets of molecular interactions with any predefined level of precision (see Figure 8). For example, we can request from a database all interactions with extraction precision 95% or greater, which would result in the case of the GeneWays 6.0 database in recall of 77.9% (Text S1 Note 13). However, we are not forced to discard the unrequested lower-than-threshold-precision interactions, as we must the chaff from the wheat in the epigraph to this article. Intuitively, even weakly supported facts (i.e., those on which there is not full agreement) can be useful in interpreting experimental results, and may gain additional support when studied in conjunction with other related facts (see Figure 1 for examples of weakly supported yet useful facts, such as *cocaine stimulates prolactin,* with a low extraction confidence, but biologically plausible; the accuracy predictions were computed using the MaxEnt 2 method). We envision that, in the near future, we will have computational approaches, such as probabilistic logic, that allow us to use weakly supported facts for building a reliable model of molecular interactions from unreliable facts (paraphrasing John von Neumann's “synthesis of reliable organisms from unreliable components” [21]).

Experiments with any standalone set of data generate results insufficient to allow us to draw conclusions about the general performance of different classifiers. Nevertheless, we can speculate about the reasons for the observed differences in performance of the methods when applied to our data. The modest performance of the Naïve Bayes classifier is unsurprising: we know that many pairs of features used in our analysis are highly or weakly correlated (see Figures 2 and 7). The actual feature dependencies violate the method's major assumption about the conditional independence of features. MaxEnt 1, which performed significantly more accurately than the Naïve Bayes in our experiments, but was not as efficient as other methods, takes into account only the class-specific mean values of features; it does not incorporate parameters to reflect dependencies between individual features. This deficiency of MaxEnt 1 is compensated by MaxEnt 2, which has an additional set of parameters for pairs of features leading to a markedly improved performance (Text S1 Note 14).

Our explanation for the superior performance of the MaxEnt 2 algorithm with respect to the remainder of the algorithms in the study batch is that MaxEnt 2 requires the least parameter tweaking in comparison with other methods of similar complexity. Performance of the Clustered Bayes method is highly sensitive to the definition of feature clusters and to the way we discretize the feature values—essentially presenting the problem of selecting an optimal model from an extensive set of rival models, each model defined by a specific set of feature clusters. Our initial intuition was that a reasonable choice of clusters can become clear from analysis of an estimated feature-correlation matrix. We originally expected that more highly correlated parameters would belong to the same cluster. However, the correlation matrices estimated from the complete GeneWays 6.0 database and from a subset of annotated facts turned out to be rather different—see Figure 2—suggesting that we could group features differently. In addition, analysis of mutual information between the class of a statement and pairs of features (see Figure 7) indicated that the most informative pairs of features are often only weakly correlated. It is quite likely that the optimum choice of feature clusters in the Clustered Bayes method would lead to a classifier performance accuracy significantly higher than that of MaxEnt 2 in our study, but the road to this improved classifier lies through a searching an astronomically large space of alternative models.

Similar to optimizing the Clustered Bayes algorithm through model selection, we can experiment with various kernel functions in the SVM algorithm, and can try alternative designs of the artificial neural network. These optimization experiments are likely to be computationally expensive, but are almost certain to improve the prediction quality. Furthermore, there are bound to exist additional useful classification features waiting to be discovered in future analyses. Finally, we speculate that we can improve the quality of the classifier by increasing the number of human evaluators who annotate each datapoint in the training set. This would allow us to improve the gold standard itself, and could lead to development of a computer program that performs the curation job consistently at least as accurately as an average human evaluator.

## Supporting Information

Text S1 Supplementary Text (47 KB PDF)

## Abbreviations

QDA: Quadratic Discriminant Analysis
Rbf: radial basis function
*ROC*: receiver operating charecteristic
SVM: support vector machines
