# Supplementary material for: Imitating Manual Curation of Text-Mined Facts in Biomedicine
Source: PLoS Comput Biol. 2006 Sep 8;2(9):e118. doi: 10.1371/journal.pcbi.0020118 (PMC1560402; doi:10.1371/journal.pcbi.0020118)
Supplement: Supplementary file 1 [file pcbi.0020118.sd001.pdf]

Supplementary Text

July 26, 2006

Notes

Note 1

The current version of GeneWays database contains $4,035,759$ redundant interactions ($2,652,916$ of them are unique) that involve $1,299,146$ unique substance terms ($102,042,092$ redundant terms were identified in total) from $232,265$ full-text articles representing 78 major research journals. The spectrum of relations represented in the database is shown in Figures 5 and 9.

Note 2

We also computed the κ-score for the inter-annotator agreement in the following way.

$$\kappa = \frac{P(A) - P(E)}{1 - P(E)}, \tag{1}$$

where $P(A) = 0.92$ is the observed pair-wise agreement between annotators, $P(E)$ is the expected agreement under the random model (so long as we have a binary classification task, we assumed $P(E) = \frac{1}{2}$), which gives $\kappa = 0.84$ for the high-agreement chunks and $\kappa = 0.48$ for the low-agreement chunk, see [Suppl. Citation 1] for guidelines on usage and interpretation of κ-values. If we use a more sophisticated random model, accounting for the observation that in our study an average evaluator assigned the label *correct* with probability 0.65 rather than 0.5, we obtain $P(E) = 0.65^2 + 0.35^2 = 0.545$, which leads to slightly lower κ-estimates of 0.82 and 0.43, for the high- and low-agreement chunks, respectively.

Note 3

The low-agreement chunk was produced by only two evaluators. We interpreted the low agreement as an indication that the evaluators, while working on this chunk, were less careful than usual, and treated this data set in the same way as an experimentalist would treat a batch of potentially compromised experiments or expired reagents.

We also considered a hypothesis that the offending chunk happened to be more difficult for evaluation than the high-agreement chunks. However, we did not find any evidence supporting this hypothesis: various semantic relation types were included in every chunk in essentially identical proportions.

Note 4

The actual scoring is slightly more complicated because a small portion of annotations provided by experts belonged to the class *uncertain* (corresponding to the option for evaluators “Unable to decide”), which was viewed as an intermediate between classes *correct* and *incorrect*—such annotation received a score of 0.

Note 5

We used 68 features, most of which are non-binary, see Table 5.

Note 6

The only exception occurs when the features are truly conditionally independent of one another. In this special case both methods should have an identical performance. The same reasoning applies to all other approximations of the full Bayesian analysis (Clustered Bayes, Discriminant Analysis, and Maximum Entropy methods): They should perform less accurately than the full Bayesian analysis whenever their assumptions are not matched exactly by the data, and perform identically to the full Bayesian analysis otherwise.

Note 7

These assumptions lead to a linear optimal decision boundary, as reflected by the name of the method.

Note 8

This change in assumptions leads to a quadratic optimal decision boundary.

Note 9

For example, if the original space is two-dimensional $\mathbf{x} = (x_1, x_2)$ and the degree d of the polynomial kernel is 2, the implicit transformation is $h(\mathbf{x}) = (1, \sqrt{2}x_1, \sqrt{2}x_2, x_1^2, x_2^2, \sqrt{2}x_1x_2)$.

Note 10

That is, we obtained estimates of the prior probabilities of classes in Equation 2, $P\left(C = c_{correct}\right) = 0.65$ and $P\left(C = c_{incorrect}\right) = 0.35$. The raw precision that we report here is much lower than estimates that we reported in earlier studies. At least three factors contributed to this discrepancy. First, we performed previous evaluations for individual components of the system, rather than over the whole text-mining pipeline. Second, after we performed our previous evaluation more than 2 years ago, we expanded substantially the list of relation types/verbs handled by the system; the most recently added relations clearly contributed to the increased error rate. Third, we performed the earlier evaluations using data sets that were at least two orders of magnitude smaller than those reported in the present study. In addition, these smaller data sets were generated by sampling *the most popular* of the extracted facts—these more popular statements probably tend to be easier to extract correctly automatically.

Note 11

Note that this way of constructing a gold standard is somewhat controversial because addition of MaxEnt 2 evaluations to the human data creates many tie evaluations (two “correct” and two “incorrect” labels for the same statement) that we later exclude from the analysis.

Nevertheless, if we were to use results produced by four human evaluators rather than three humans and a program, we would create the four-evaluator gold standard in exactly the same way.

Note 12

Alan M. Turing proposed an experiment for testing machine intelligence by interrogating the machine and a group of humans through a mediator, the goal being to distinguish the humans from the machine on the basis of only typewritten replies. If the interrogator fails to make the correct distinction, machine intelligence passes the test. Applying the Turing test to our problem, we imagine that we have a group of four evaluators, one of which is a computer. If we cannot single out the computer-embodied evaluator on the basis of a higher error rate, our artificial evaluator passes the test. Obviously, we mention the Turing test only as a metaphor — we are not claiming that MaxEnt 2 would pass the actual Turing test.

Note 13

Recall is defined as $\frac{\textit{number of true positives}}{\textit{number of true positives}+\textit{number of false negatives}}$. The false-negative results—the facts that were in the original text but were missed by the system—can be generated at two stages of the analysis. The first stage, information extraction, occurs when the GeneWays pipeline recovers facts with recall β—we did not try to measure the value of β in the present study. The second stage is the automated curation of the database, during which all facts with score below a certain threshold are discarded. This second stage is associated with an additional loss of the recall: Only a proportion, ρ, of the originally extracted true-positive facts is retained. It is the second type of recall that we discuss in the text (see Figure 8). The overall recall, including both stages of the analysis, is just a product of the two values, $\beta\rho$.

Note 14

We also analyzed the relation between the size of the training data set and the accuracy of MaxEnt 2 method. While accuracy of the MaxEnt 2 method with the whole training data set was 87.97%, it dropped to 87.38% when using only 60% of the training data, and to 83.57% with 20% of the training data.

Supplementary Citations

[1] Carletta J (1996) Assessing agreement on classification tasks: The kappa statistic. Computational Linguistics 22:249-254.
